# Signaling of double strand breaks and deprotected telomeres in *Arabidopsis*

**DOI:** 10.3389/fpls.2013.00405

**Published:** 2013-10-16

**Authors:** Simon Amiard, Maria E. Gallego, Charles I. White

**Affiliations:** Génétique, Reproduction et Développement, UMR CNRS 6293/U1103 INSERM/Clermont Université, Université Blaise PascalAubiére cedex, France

**Keywords:** signaling, sensing, double strand breaks, telomere, DNA repair

## Abstract

Failure to repair DNA double strand breaks (DSB) can lead to chromosomal rearrangements and eventually to cancer or cell death. Radiation and environmental pollutants induce DSB and this is of particular relevance to plants due to their sessile life style. DSB also occur naturally in cells during DNA replication and programmed induction of DSB initiates the meiotic recombination essential for gametogenesis in most eukaryotes. The linear nature of most eukaryotic chromosomes means that each chromosome has two “broken” ends. Chromosome ends, or telomeres, are protected by nucleoprotein caps which avoid their recognition as DSB by the cellular DNA repair machinery. Deprotected telomeres are recognized as DSB and become substrates for recombination leading to chromosome fusions, the “bridge-breakage-fusion” cycle, genome rearrangements and cell death. The importance of repair of DSB and the severity of the consequences of their misrepair have led to the presence of multiple, robust mechanisms for their detection and repair. After a brief overview of DSB repair pathways to set the context, we present here an update of current understanding of the detection and signaling of DSB in the plant, *Arabidopsis thaliana*.

## DSBs REPAIR PATHWAYS IN *ARABIDOPSIS THALIANA*

Double strand breaks (DSB) repair pathways are classed as either homologous recombination (HR) or non-homologous end-joining (NHEJ), depending upon the dependence or not on DNA sequence homology between the recombining molecules. HR requires the presence of an intact homologous DNA template and is most active in S/G2 phase when the sister chromatid is present. The critical step during HR is the formation of RAD51 filament on the 3′ ended single-stranded DNA (ssDNA) produced by resection of the breaks. The nucleofilament formed by RAD51 on the broken DNA molecule catalyzes the invasion of a homologous DNA template sequence by the 3′ ended DNA strand(s), which are extended through DNA synthesis, and finally the joint recombination intermediate is resolved to complete the process (for review, Heyer and Liu, 2010). The major players in HR are very highly conserved and most have been identified and characterized in *Arabidopsis thaliana *(Mannuss et al., 2011).

The participation, or not, of the KU complex permits classification of NHEJ pathways into two categories: direct joining of breaks through the KU-dependent pathway and end-joining involving microhomologies by the KU-independent microhomology-mediated (MMEJ) and “alternative” or “back-up” end-joining (Alt-NHEJ or B-NHEJ; for review, Decottignies, 2013). In *Arabidopsis* the KU-dependent pathway has been the subject of a number of studies (Riha et al., 2002; Friesner and Britt, 2003; Gallego et al., 2003; Van Attikum et al., 2003). The distinction between KU-independent pathways is not clear because both imply the use of microhomology sequence to repair the break. In vertebrates, it is known that Alt-NHEJ is based on the action of proteins usually known for their role in single strand breaks repair XRCC1, PARP1 and LIG3 (Decottignies, 2013). In *Arabidopsis*, the conservation of this pathway has been confirmed through studies of XRCC1 (Charbonnel et al., 2010) and PARP1/PARP2 (Jia et al., 2013). Concerning the MMEJ pathway, the first actors identified were the MRX (MRN) and the Rad1/Rad10 (ERCC1/XPF) complexes in yeast (Ma et al., 2003). Similarly in *Arabidopsis*, MRE11 has been implicated in the use of microhomologies in telomere fusions (Heacock et al., 2004) and XPF has been shown to be involved in a third NHEJ pathway of DSB repair independent of the KU complex and XRCC1 (Charbonnel et al., 2011).

The viability of the single and multiple mutants for each of these pathways in *Arabidopsis* permitted study of the kinetics of DSB repair *in planta*, establishing a hierarchy of DSB repair pathways in *Arabidopsis* (Charbonnel et al., 2011). A surprising result of this study was the ability of quadruple *ku80 xrcc1 xpf xrcc2* mutants (invalidated for all known HR and NHEJ pathways) to repair ionising radiation (IR)-induced DSB, but at a very reduced rate. Although this “repair” is accompanied by high levels of anaphase chromosome bridging, plants cells are thus able to repair DSB in the absence of all four major DSB repair pathways. This results points to another end-joining pathway that would be activated in case of extreme stress and could be one part of the explanation of the striking ability of plants to develop in presence of high levels of genome damage.

The choice of repair mechanisms is tightly regulated with respect to the cell cycle phase and the nature of the break (Chapman et al., 2012). DSB end resection has been shown to be an essential step for the choice of repair pathway, with recent reports showing the implication of 53BP1-RIF1 in blocking resection and thus stimulating NHEJ, and BRCA1-CtIP promoting DNA resection and HR in mammals (Chapman et al., 2013; Escribano-Díaz et al., 2013; Zimmermann et al., 2013). CtIP (Uanschou et al., 2007) and BRCA1 (Lafarge and Montane, 2003; Trapp et al., 2011) orthologs, but not 53BP1 or RIF1, have been described in *Arabidopsis*, but no detail of their roles in these processes have been reported.

ncRNA (non-coding RNA) are clearly involved in multiple aspects of DNA repair. miRNA (microRNA) transcription is induced after DNA damage and these small RNA are believed to be involved in the regulation of DNA damage repair proteins (reviewed by Chowdhury et al., 2013). Recent work shows links directly to DSB repair in *Arabidopsis* as well as in mammalian cells. Small RNA (diRNA) are produced directly at break sites and are required for correct repair, probably through chromatin modifications or through the recruitment of repair proteins to facilitate repair (Wei et al., 2012).

## SIGNALING OF DSBs

The first essential step of the repair process is the recognition and the signaling of the DNA break. This step is critical as it allows cell-cycle arrest, recruitment of DSB repair proteins, chromatin remodeling and eventually cell death or senescence (Goodarzi et al., 2010). In yeast as well as in mammals, the main factors involved in the sensing of the DSB are the MRX/N (Mre11, Rad50 and Xrs2/Nbs1) and the KU (Ku70/Ku80) complexes that compete for binding to unprocessed DSBs (Hiom, 2010). Together with DNA-PKcs, the human KU complex, forming the DNA-PK holoenzyme, functions as a DNA end-bridging factor leading to repair via NHEJ, essentially in G1 phase (Lieber, 2010). In G2 phase, the binding of KU is inhibited and the MRN complex initiates repair via HR (Heyer and Liu, 2010). In plants as well as in yeast, the DNA-PKcs enzyme is not conserved, hence the tethering of the DNA ends is presumably carried out by the MRN complex or by other proteins.

The signaling role is then assumed by specific kinases belonging to the PI3K-like protein kinase family (PIKK): Tel1/ATM and Mec1/ATR. The binding of the yeast MRX complex to the DSB promotes the recruitment of Tel1 leading to Tel1-dependent cell cycle checkpoint activation prior to DNA processing (Usui et al., 2001). Absence of Tel1 can be compensated for by Mec1 (Morrow et al., 1995), with the yeast *tel1* mutant being checkpoint sufficient and not hyper-sensitive to DNA damaging agents (Mantiero et al., 2007). In vertebrates, ATM is activated by DNA double-strand breaks, while ATR is activated by ssDNA, formed notably in processing blocked replication forks (Cimprich and Cortez, 2008). Once bound to DNA, MRN recruits and activates ATM via interaction with Nbs1 (Lavin, 2007) and Mre11 nuclease activity leads to the formation of single strand oligonucleotides that further promote ATM activation (Jazayeri et al., 2008). Further maturation of the DNA extremities can also lead to ssDNA formation and ATR activation (Jazayeri et al., 2006). Mutation of ATM in humans leads to Ataxia-telangectasia (A-T), a genomic instability disorder characterized by neurodegeneration, immunodeficiency and sensibility to ionizing radiation. At the cellular level, the hallmarks of ATM deficiency are increased chromosomal breakage and premature senescence (Shiloh and Ziv, 2013). In the absence of ATM (in A-T cells), signaling of DNA breaks can be accomplished by ATR helped by EXO1, however, the absence of both kinases results in the absence of cell cycle arrest due to defects in signaling of breaks (Tomimatsu et al., 2009).

Mec1/ATR is considered to be the specific sensor of DNA replication fork stalling and DNA replication damage, and is more generally activated by a variety of lesions that have in common the generation of ssDNA. Irrespective of the origin of the ssDNA, ATR is recruited by its cofactor ATRIP, which indirectly recognizes ssDNA through interaction with the ssDNA-binding protein, RPA. The 9-1-1 checkpoint clamp has also been implicated in activation of the ATR/Mec1 kinase (Majka et al., 2006). Mec1 is an essential gene in yeast (Weinert et al., 1994) and even in the absence of exogenous genotoxic stress, Mec1 mutants accumulate gross spontaneous chromosomal rearrangements (GCRs; Myung and Kolodner, 2002). ATR deficiency is lethal in mammalian cells but hypomorphic *atr* mutations have been described in a few patients with the rare Seckel syndrome, characterized by microcephaly and growth retardation (O’Driscoll et al., 2003).

The presence of ATM and ATR is well conserved while, as for yeast, no DNA-PK ortholog has been identified in plants. IR-induced gamma-H2AX foci are mediated essentially by ATM and less so by ATR, with no foci observed in irradiated *atm atr* mutant cells (Friesner et al., 2005), confirming that AtATM and AtATR are the only DSB signaling PIKK kinases in plants. The presence in *Arabidopsis* of the protein AtATRIP, necessary for AtATR activation as seen in mammals, further reinforces the idea that DNA damage signaling in plants is conserved (Sweeney et al., 2009). The role of the MRN complex in DNA damage detection and activation of kinase mediated signaling is conserved in *Arabidopsis* (Amiard et al., 2010) and plant homologs of the genes encoding the 9-1-1 (Rad9/Rad1/Hus1) sensor complex have been identified and are required for resistance to the DNA damaging agents Bleomycin and Mitomycin C (MMC; Heitzeberg et al., 2004).

*Arabidopsis* *atm* mutants are phenotypically wild type, except for a partial sterility (Culligan and Britt, 2008). These plants are however hypersensitive to ionizing irradiation and methyl methane sulphonate (MMS), but not to UV irradiation. *Arabidopsis* *atr* mutants are viable, fertile, and like *atm* mutants, phenotypically wild-type in the absence of exogenous DNA damaging agents. *atr* mutants are hypersensitive to hydroxyurea and aphidicolin, due to a defective G2 checkpoint response to blocked replication forks (Culligan et al., 2004). ATR can however partially compensate for the ATM response, as the double *atm atr* mutant is completely sterile due to meiotic prophase genome fragmentation (Culligan and Britt, 2008).

Neither ATR nor ATM signaling is thus essential during normal plant development – a surprising result given the conservation of the roles of these proteins in plants and the lethality of the corresponding mutants in mammals. A hint to a possible explanation for this could come from the ability of DSBs to be repaired in plants in the double *rad50 atr* mutant, which combines absence of ATM and ATR activities and absence of H2AX phosphorylation (see next section). Spontaneous DSBs appear in consequence of replication defects in these plants and result in high levels of anaphase bridging, showing that *Arabidopsis* can repair DSB in the absence of PIKK activation (Amiard et al., 2010).

Once activated, PIKK can activate many targets necessary to maintain genomic integrity (Culligan et al., 2006; Matsuoka et al., 2007). Phosphorylation of the histone variant H2A/H2AX around the break by PIKK is an early cellular response to the induction of DSBs and occurs over 50 kb in yeast to 2 Mb for H2AX in mammals. H2AX phosphorylation is easily detected using phospho-specific antisera and has emerged as a highly specific and sensitive molecular marker for monitoring DNA damage and its repair (Kinner et al., 2008). Although not required for the initial recruitment of signaling and repair factors, H2AX phosphorylation is essential for their accumulation at the breaks (Celeste et al., 2003; Fernandez-Capetillo et al., 2003; Fillingham et al., 2006). The importance of this is seen in the sensitivity to DSB damaging agents, impaired DSB repair and defects in G1 checkpoint activation of yeast mutants of the H2A gene (Downs et al., 2000; Redon et al., 2003; Hammet et al., 2007) and similar phenotypes of mammalian cells and mice deficient for H2AX (Celeste et al., 2002). Moreover H2AX deficient mice were radiation sensitive, growth retarded, immune deficient and males were infertile.

In contrast, *Arabidopsis* mutants for this histone develop normally and only a slight defect in DSB repair has been reported in RNAi knock-down lines (Lang et al., 2012). Moreover, the phosphorylation of this histone does not seem required for DSB repair in plants, as seen in the chromosome fusions observed in the *rad50 atr* double mutant (Amiard et al., 2010). This being so, how is DSB signaling mediated in the absence of H2AX phosphorylation in *Arabidopsis*? A possible answer comes from reports showing roles of modifications of other histones around DSB in mammals: ubiquitinylation of H2A by RNF8 is required for proper 53BP1 recruitment (Marteijn et al., 2009; Rossetto et al., 2010) and a role for histone lysine methylation in DSB repair is supported by the observation that H3K36me2 enhances DNA repair by NHEJ (Fnu et al., 2011). H3K36me2, once formed at DSB site, may create docking sites for other repair proteins, recruiting them for transcription and DNA repair. It will be of great interest to see whether such modifications also play important roles in repair of DSBs in plants.

## SIGNALING OF DEPROTECTED TELOMERES

Telomeres consist of an elaborate, higher-order assembly of specific DNA sequence and proteins that cooperatively provide protection against degradation and recombination of the ends of linear eukaryotic chromosomes. In vertebrates, telomere protection is provided mainly by Shelterin, a complex of six telomeric proteins (TRF1, TRF2, POT1, TIN2, TPP1 and RAP1) that prevents inappropriate recombination and fusion between telomeres, and also has complementary roles in telomere replication and length regulation (Palm and De Lange, 2008; Martínez and Blasco, 2011). TRF1 and TRF2 bind to the duplex region of the telomere and searches for TRF-like proteins in *Arabidopsis* have identified many proteins able to bind double-stranded telomeric DNA (Zellinger and Riha, 2007; Amiard et al., 2011b; Peška et al., 2011). None of these seems however to be essential for telomere protection, suggesting redundancy of double-stranded DNA binding telomeric proteins in plants. POT1 binds to the natural single-stranded (ss) extension of the G-rich strand of chromosome ends (G-overhang or 3′-overhang) and in both humans and *Saccharomyces* pombe, POT1 plays a key role in telomere end protection (Baumann and Cech, 2001). *Arabidopsis* has two POT1 orthologs, POT1a and POT1b, both of which associate with the telomerase ribonucleoprotein but do not bind telomeric ssDNA and are not essential for telomere capping (Surovtseva et al., 2007; Shakirov et al., 2009; Cifuentes-Rojas et al., 2011).

In *S. cerevisiae* there has been no shelterin-like complex identified to date and a somewhat simpler protection complex, consisting mainly of the CST complex (Cdc13, Stn1 and Ten1), is present (Garvik et al., 1995; Grandin et al., 2001; Shore and Bianchi, 2009). Yeast Cdc13, together with Stn1 and Ten1, plays a dual role in telomere end protection and regulation of telomere replication. Orthologs of the* S. cerevisiae *CST proteins have been found in humans and mouse, as well as in *Arabidopsis* (Miyake et al., 2009; Surovtseva et al., 2009). Recent studies in mammalian cells reveal that the CST complex seems to be implicated in facilitating telomere replication by rescuing replication after fork stalling (Stewart et al., 2012) and that this complex is involved in the regulation of the telomeric 3′ overhang by C-strand fill-in by Polymerase alpha (Wang et al., 2012). Plants appear to represent an evolutionary intermediate between *S. cerevisiae*, which has only CST as a capping complex, and vertebrates which use both shelterin and CST complex for telomere capping and correct telomeric replication (Giraud-Panis et al., 2010; Price et al., 2010).

Deprotected telomeres are recognized by cells as DSB, and their “repair” results in chromosome fusions/rearrangements and genomic instability (De Lange, 2009). As for other DSB, deprotected telomeres are substrates for kinase activation and are characterized by the appearance of TIFs (telomere induced foci), DNA damage response factors that coincide with telomere signals. In mammals the absence of TRF2 or POT1 leads to the appearance of TIFs and this depends upon ATM and ATR, respectively (De Lange, 2009). In plants, we have shown that the appearance of TIFs in *ctc1* or *stn1* mutants are exclusively ATR-dependent and that in absence of the catalytic subunit of the telomerase (TERT), the short deprotected telomeres are recognized as DSBs through the activation of both ATM and ATR (Amiard et al., 2011a). Surprisingly, we have shown that in the *Arabidopsis* *ctc1 atr* mutant, which does not form TIFs, telomeres are still able to fuse. This result contrast clearly with the situation in vertebrates, where ATM and ATR are absolutely required for fusion of deprotected telomeres in absence of the TRF2 or POT1, respectively (Denchi and De Lange, 2007). Hence here again, plant repair pathways can still be activated in absence of the kinase activity.

## CONCLUSION

This short review summarizes knowledge concerning DNA break signaling in *Arabidopsis thaliana*. A list of genes discussed here is presented in **Table 1** and we refer interested readers to a recent compilation of *Arabidopsis* DNA repair/recombination genes (). Given the crucial importance of the signaling step in DNA repair it is not surprising to find strong conservation of these mechanisms in higher eukaryotes. Nevertheless, evidence points to a particular ability of plants to repair even in absence of signaling and the presence of an unknown plant specific repair pathway(s) is now suspected. Plants possess a not fully understood ability to resist and develop in presence of DNA damaging agents and the implication of plant specific recombination events could provide part of the explanation for this. The increased spontaneous recombination rates seen in plants subjected to biotic or abiotic stresses (review by Waterworth et al., 2011) has been proposed to be a programmed response increasing the plasticity of plant genome leading to acceleration of plant evolution (Molinier et al., 2006; Boyko and Kovalchuk, 2011).

**Table 1:** Major factors involved in DNA double strand break signaling and repair and telomere protection in budding yeast, human and *Arabidopsis thaliana*.

	*Saccharomyces cerevisiae*	Human	*Arabidopsis thaliana*
Sensing	Mre11/Rad50/Xrs2	Mre11/Rad50/Nbs1	Mre11/Rad50/Nbs1
Signaling	Mec1	ATR	ATR
	Tel1	ATM	ATM
	H2A	H2AX	H2AX
**Mediators**
ATM	Rad9	53BP1	n.i.
signaling	Rif1	RIF1	n.i.
	n.o.	BRCA1	BRCA1
	n.o.	BRCA2	BRCA2
	Sae2	CtIP	COM1
ATR	Ddc2	ATRIP	ATRIP
signaling	Ddc1/Rad17/Mec3	RAD9/RAD1/HUS1	RAD9/RAD1/HUS1
	Rfa	RPA	RPA
HR	Rad51	RAD51	RAD51
	Rad51 paralogs: (Rad55/Rad57/Shu1/Shu2/Csm2/Psy3)	RAD51 paralogs: (RAD51B/C/D/XRCC2/XRCC3)	RAD51 paralogs: (RAD51B/C/D/XRCC2/XRCC3)
	Rad52	RAD52	RAD52 (2 genes)
	Rad10	ERCC1	ERCC1
	Rad1	XPF	XPF
	Exo1	EXO1	EXO1
NHEJ	Ku70/Ku80	KU70/KU80	KU70/KU80
	Dnl4	LIG4	LIG4
	Lif1	XRCC4	XRCC4
	n.o.	XRCC1	XRCC1
	n.o.	PARP1	PARP1
	n.o.	PARP2	PARP2
	n.o.	LIG3	n.i.
	n.o.	DNA-PKcs	n.o.
Telomeric protection	n.o.	TRF1	n.i.
	n.o.	TRF2	n.i.
	n.o.	POT1	POT1A/POT1B
	n.o.	TIN21	n.i.
	n.o.	TPP1	n.i.
	Rap1	RAP1	n.i.
	Cdc13	CTC1	CTC1
	STN1	STN1	STN1
	TEN1	TEN1	TEN1

*n.o., no ortholog; n.i., no identified orthologe reported.*

## Conflict of Interest Statement

The authors declare that the research was conducted in the absence of any commercial or financial relationships that could be construed as a potential conflict of interest.
